# *Legionella pneumophila* in Rainwater on Roads

**DOI:** 10.3201/eid1508.090317

**Published:** 2009-08

**Authors:** Ryota Sakamoto, Akira Ohno, Toshitaka Nakahara, Kazunari Satomura, Suketaka Iwanaga, Yuuichiro Kouyama, Fumiaki Kura, Naoyuki Kato, Kozo Matsubayashi, Kiyohito Okumiya, Keizo Yamaguchi

**Affiliations:** Kyoto University, Kyoto, Japan (R. Sakamoto, T. Nakahara, K. Satomura, S. Iwanaga, K. Matsubayashi); Toho University School of Medicine, Tokyo, Japan (A. Ohno, Y. Kouyama, N. Kato, K. Yamaguchi); National Institute of Infectious Diseases, Tokyo (F. Kura); Research Institute for Humanity and Nature, Kyoto (K. Okumiya)

**Keywords:** Legionnaires’ disease, Legionella pneumophila, rain, bacteriology, bacteria, Japan, dispatch

## Abstract

During rain, transient puddles form on roads, and this water is splashed into the air by moving vehicles. To determine whether this water contains *Legionella* *pneumophila,* we collected samples from roads. We found that *L. pneumophila* are abundant in these puddles, especially during warm weather.

*Legionella* *pneumophila* bacteria are a major cause of severe community-acquired pneumonia; in recent years the numbers of reported cases of legionellosis have increased substantially where testing is available (e.g., United States, Europe, Japan). However, the source and mode of transmission for sporadic cases are often obscure. Several studies have indicated an association between rain and legionellosis (*1*–*5*). We recently reported a case of legionellosis in a commercial truck driver in Japan who became infected during the rainy season (*6**,**7*). No particular environmental risk factors were noted, although he had certain host risk factors such as middle age (mid 50s) and heavy smoking. All specimens collected from his home environment had negative *L. pneumophila* culture growth.

In this study, we investigated the prevalence of *L. pneumophila* in puddles on asphalt roads. Our hypothesis was that after rain *L. pneumophila* grows prolifically in puddles on asphalt roads and can be spread by moving cars, which may increase the amount of organism in the environment.

## The Study

From July through October 2007, we collected 45 samples of rainwater from 4 points on 1 asphalt road in Tokyo, Japan. After rainfall, 100-mL water samples were suctioned from road surfaces into sterile syringes and then stored in sterile bottles. Also, sterile flat containers were placed beside the roads for 1 day so that raindrops were collected directly. Swabs from roads were also collected when it was sunny. Meteorologic data for the sample collection dates were obtained from the Japan Meteorological Agency. Water samples were concentrated and mixed with equal volumes of 0.2 M KCl–HCl buffer (pH 2.2). The mixture was allowed to stand at room temperature for 15 min before being spread onto WYOα plates (Eiken Chemical Co., Tokyo, Japan). The plates were then incubated for at least 7 days. Smooth colonies that were grayish white or gray-blue-purple were subcultured on *Legionella* agar (Becton-Dickinson, Mountain View, CA, USA) supplemented with l-cysteine and ferric pyrophosphate and on nutrient agar (Eiken Chemical co.). Colonies that grew on only *Legionella* agar were subsequently identified by PCR. The following primers were used to detect the 430-bp sequence of the 16S r RNA-encoded gene: forward primer 5′-GAGGGT TGATAGGTTAAGAGC-3′ and reverse primer 5′-CGGTCA ACT TATCGCGTT TGCT-3′ (*8*). Serotyping was also performed by a slide agglutination test with commercial specific antiserum (Denka Seiken Co., Ltd, Tokyo, Japan). The detection limit of the procedure was 20 CFU/100 mL. A loop-mediated isothermal amplification (LAMP) kit (Eiken Chemical Co.) was also used to detect *Legionella* spp. (*9*). To confirm the presence of free-living amebae (FLA) DNA, we used PCR with forward primer P-FLA-F: 5′-CGCGGTAATTCCAGCTCCAATAGC-3′ and reverse primer P-FLA-R: 5′-CAGGTTAAGGTCTCGTTCGTTAAC-3′ targeted at conserved stretches of *Acanthamoeba* 18S rDNA (*10*). For molecular typing, we used repetitive-element PCR with 18-mer degenerate primers REP1R-Dt (3′-CGGNCTACNGCNGCNIII-5′) and REP2-Dt (3′-CATCCGGNCTATTCNGCN-5′) (N = A, C, G, and T; I = inosine) (*11*).

**Figure 1:**
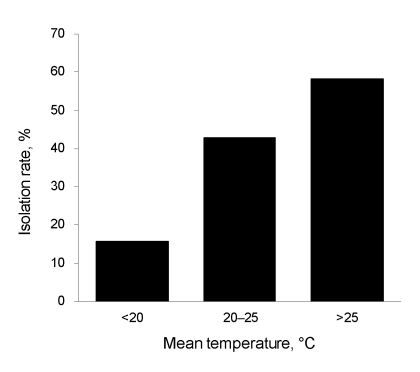
Isolation rate (%) of *Legionella pneumophila* according to mean environmental temperature on sampling date, Tokyo, Japan.

**Figure 2:**
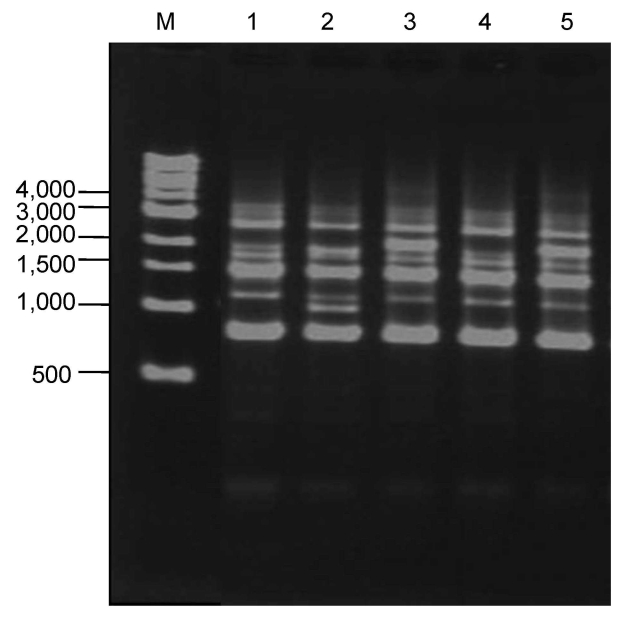
Repetitive-element PCR DNA fingerprints of *Legionella pneumophila* serogroup 1 isolates (lanes 1–5) from the same puddle of rainwater on an asphalt road in Tokyo, Japan. Lane M shows DNA reference marker sizes (New England BioLabs, Inc., Ipswich, MA, USA) in basepairs.

We took samples from puddles at 18 points as a primary survey. Simultaneously, as fixed-point observations, we set 4 points on a road and collected samples when puddles were formed. In the primary survey, *L. pneumophila* were detected by culture in 7 of 18 samples taken from puddles. *Acanthamoeba* spp. were detected by PCR in 4 of 18 puddles. *L.* *pneumophila* were not detected by culture of swab samples obtained from asphalt surfaces on sunny days, although they were detected in 2 of 12 samples tested by the LAMP method. *Acanthamoeba* spp. were also detected by PCR from 2 of 12 swab samples (Table). In the fixed-point observations, 16 (35.6%) of 45 puddle samples were positive by culture. According to serogroup typing of 150 strains of *L. pneumophila* isolated, the most prevalent serogroup was serogroup 1 (n = 56 [37.3%]) (Table). *L. pneumophila* were detected by culture in 3 (15.8%) of 19 samples from puddles when mean temperature was <20°C, in 6 (42.9%) of 14 samples when mean temperature was 20°C–25°C, and in 7 (58.3%) of 12 samples when mean temperature was >25°C (p = 0.043) (Figure 1). With regard to molecular typing by repetitive-element PCR, organisms of lanes 1–5 were all detected on the same day in the same puddle and identified as *L. pneumophila* serogroup 1. Although lanes 1–3 appeared to have different patterns, lanes 1 and 4 and lanes 3 and 5 appeared indistinguishable (Figure 2).

**Table:** *Legionella* spp. and *Acanthamoeba* spp. isolated from asphalt road sources, Tokyo, Japan, July–October 2007

Source	Positive samples, no. (%)
Primary survey	
Puddles on roads (n = 18)	
L. pneumophila*	7 (38.9)
*Acanthamoeba* spp.†	4 (22.2)
Rainwater (n = 10)	
L. pneumophila*	0
*Legionella* spp.‡	1 (10.0)
*Acanthamoeba* spp.†	3 (30.0)
Swabs from roads on sunny days (n = 12)	
L. pneumophila*	0
*Legionella* spp.‡	2 (16.7)
*Acanthamoeba* spp.†	2 (16.7)
Fixed-point observation of puddles on roads	
*L. pneumophila* concentration (n = 45)	
*<*20 CFU/100 mL	29 (64.4)
20–99 CFU/100 mL	7 (15.6)
100–999 CFU/100 mL	7 (15.6)
*>*1,000 CFU/100 mL	2 ( 4.4)
*L. pneumophila* serogroups (n = 150 strains isolated)	
1	56 (37.3)
3	50 (33.3)
2	15 (10.0)
6	12 ( 8.0)
5	11 ( 7.3)
Others	6 ( 4.0)

*Tested by using culture method. †Tested by using PCR method. ‡Tested by using loop-mediated isothermal amplification method.

Isolation of *L. pneumophila* in puddles of rainwater on asphalt roads indicates that the organisms may be spread from wet roads into the air by moving vehicles. Thacker et al. reported an outbreak of legionellosis after a heavy rainstorm (*1*). In our study, *L. pneumophila* were not isolated by culture of direct rainwater samples or of swabs collected from road surfaces on sunny days, but they were detected in these 2 sample types by the LAMP method. Factors known to support colonization of *L. pneumophila* are humidity, stagnation, scales (residues, deposits), sediments, and temperature (*12*). When *L. pneumophila* are exposed to unsuitable conditions, it changes its metabolism to reduce growth activity and remains viable but not culturable. *Acanthamoeba castellanii* have been shown to resuscitate *L. pneumophila* in this viable but nonculturable state (*13*). We identified *Acanthamoeba* spp. by PCR from raindrops and from swabs of asphalt pavement surface on sunny days. In a previous study, we demonstrated that at temperatures >25ºC, *L. pneumophila* could replicate in amebae and that at temperature <20ºC, amebae digested *L. pneumophila* and eliminated them through the process of encystation (*14*). It is possible that *L. pneumophila* are in a viable but nonculturable state in raindrops in the air or that they survive on asphalt on sunny days and return to a proliferative state in rainwater on asphalt roads, especially during warm weather.

*L. pneumophila* may be splashed from puddles into surrounding air. In this regard, Fisman et al. identified an association between legionellosis and precipitation (odds ratio 2.48, 95% confidence interval [CI] 1.30–3.12) and increased humidity (odds ratio per 1% increase in relative humidity 1.08, 95% CI 1.05–1.11) 6–10 days before identification of infected patients (*3*). Hicks et al. reported that a 1-cm increase in rainfall was associated with a 2.6% (risk ratio 1.026, 95% CI 1.012–1.040) rise in incidence of legionellosis (*4*). The increase in reported legionellosis patients may result partly from growth of *L. pneumophila* in puddles on roads, although the possibility of an increase in *L. pneumophila* in tap water after rain cannot be excluded. Because airborne *L. pneumophila* can survive longer at high relative humidity (*15*), after rain the organism may be sprayed into the air and increase both on the ground and in the air.

## Conclusions

The frequent presence of *L. pneumophila* in puddles of rainwater on asphalt roads, especially during warm weather, indicates the possibility of frequent contact with *L. pneumophila–*containing aerosols. To decrease illness and death from legionellosis, community physicians should consider the possibility of legionellosis for pneumonia patients, even those who had not traveled or visited spa facilities, especially during warm, rainy weather. To prevent legionellosis, persons must consider their risk factors; *L. pneumophila* may be more ubiquitous than previously thought.
